# Molecules and fossils reveal punctuated diversification in Caribbean “faviid” corals

**DOI:** 10.1186/1471-2148-12-123

**Published:** 2012-07-25

**Authors:** Sonja A Schwartz, Ann F Budd, David B Carlon

**Affiliations:** 1 Department of Environmental Science, Policy & Management, University of California, Berkeley, CA, 94720, USA; 2 Department of Geoscience, University of Iowa, Iowa City, IA, 52242, USA; 3 Department of Biology, University of Hawaii at Manoa, Honolulu, HI, 96822, USA

**Keywords:** Scleractinia, Speciation, Adaptive radiation, Miocene, Pliocene, Coral reef

## Abstract

**Background:**

Even with well-known sampling biases, the fossil record is key to understanding macro-evolutionary patterns. During the Miocene to Pleistocene in the Caribbean Sea, the fossil record of scleractinian corals shows a remarkable period of rapid diversification followed by massive extinction. Here we combine a time-calibrated molecular phylogeny based on three nuclear introns with an updated fossil stratigraphy to examine patterns of radiation and extinction in Caribbean corals within the traditional family Faviidae.

**Results:**

Concatenated phylogenetic analysis showed most species of Caribbean faviids were monophyletic, with the exception of two *Manicina* species. The time-calibrated tree revealed the stem group originated around the closure of the Tethys Sea (17.0 Ma), while the genus *Manicina* diversified during the Late Miocene (8.20 Ma), when increased sedimentation and productivity may have favored free-living, heterotrophic species. Reef and shallow water specialists, represented by *Diploria* and *Favia*, originate at the beginning of the Pliocene (5 – 6 Ma) as the Isthmus of Panama shoaled and regional productivity declined.

**Conclusions:**

Later origination of the stem group than predicted from the fossil record corroborates the hypothesis of morphological convergence in *Diploria* and *Favia* genera. Our data support the rapid evolution of morphological and life-history traits among faviid corals that can be linked to Mio-Pliocene environmental changes.

## Background

Explaining rapid diversification and speciation remains a central challenge to evolutionary biology [1,2]. Much work has focused on either understanding the ecology and phylogenetic history of species-rich systems that have recently diversified along ecological axes (e.g. adaptive radiations) [3], or looking for patterns of species change in the fossil record [4-8]. Taking the molecular phylogenetic/ecological approach alone, however, excludes information about extinct lineages that may substantially bias our ability to identify cases of rapid diversification [9]. Conversely, relying on the fossil record alone limits our ability to detect evolutionary relationships between fossil taxa and some shifts in ecological function that may not be apparent from fossil character states. Ultimately, a more complete understanding of the processes that drive rapid diversification will require historical information from both molecular and fossil data. By examining systems that show recent speciation within monophyletic groups, ecological differentiation, and a strong fossil record, we can begin to link past to present processes in the understanding of the evolution of diversity.

The marine Caribbean fauna provides rare examples of diversification of monophyletic lineages within the context of well-understood changes in biogeography, oceanography, and climate. The isolation of Caribbean populations from their Indo-Pacific counterparts started ~15-17 Ma when the closure of the Tethys Sea cut off connections between the Mediterranean and Indo-Pacific [10]. Isolation was complete ~3.45 - 4.25 Ma when the rise of the Isthmus of Panama severed all Caribbean connections to the Indo-Pacific [11]. The period leading up to closure of the isthmus during the late Miocene to late Pliocene was characterized by changing global oceanographic circulation patterns, leading to drastic environmental, ecological, and taxonomic shifts within the Caribbean basin. Not only did the cessation of gene flow between the Pacific and Atlantic Oceans lead to widespread vicariant speciation across the newly formed isthmus [12-14], but on the Caribbean side, the accompanying geological and oceanographic changes caused an overall decrease in depth, primary productivity and turbidity and an increase in salinity, temperature, and local environmental heterogeneity [11,15]. Fossil records of many marine taxa during this period show elevated levels of taxonomic turnover [11,16-21], suggesting that climatic and geological variables drove elevated rates of cladogenesis and extinction.

This taxonomic turnover is particularly striking in corals of the family Faviidae, where an examination of the stratigraphic ranges shows that all extant species originated nearly simultaneously during the Mio-Pliocene [22]. Moreover, for faviids, this recent radiation has resulted in impressive diversification of ecological and life-history traits [23,24]. Modern species of *Manicina* are representative of a free-living lifestyle adapted to sediment-rich seagrass habitats that expanded during the Miocene then contracted during the Plio-Pleistocene [15]. In contrast, species of the brain coral genus *Diploria* tend to be reef-builders, dominating shallow water reef platforms in Pleistocene and modern times [25-28]. These two “sediment” and “reef” clades appear to share a common ancestor and ecological diversification seems to have occurred over a short period of geological time, suggesting it is tied to the contemporaneous increase in environmental heterogeneity [29]. Yet this punctuated diversification event is inferred from a fossil record, which may be incomplete or contain uncertainties in dating and taxonomic relationships that may influence our interpretation of past patterns.

Molecular data combined with well sampled fossil records provide opportunities to test existing evolutionary hypotheses and extend our understanding of both the tempo and mode of evolutionary diversification. In the Scleractinia, deep divergences between coral orders, suborders and families are increasingly well understood [30-33]. Yet a recent series of phylogenies exploring relationships at the familial level and below have demonstrated pervasive polyphyly and paraphyly at the generic level [34-39]. In addition, these studies have shown that between ocean basins, species group geographically rather than taxonomically [35,38,39]. In particular, Atlantic lineages of Faviidae and Mussidae appear to be more closely related to other Atlantic lineages than to congeners or even confamilials in other ocean basins. This geographic split supports the evidence from the fossil record of a radiation of the Caribbean coral fauna before complete isolation from the Pacific. However, the failure to resolve species relationships within the traditional coral family Faviidae, and a long history of taxonomic difficulties in identifying and classifying corals [32,36,40] demands an independent assessment of trends apparent in the fossil record.

To explore the tempo and mode of this evolutionary diversification, we unite a new multi-locus phylogeny of the Caribbean Faviidae with new stratigraphic compilations from the fossil record. Our well-sampled phylogeny allows Bayesian approaches to place these relationships into a temporal context by dating divergence times based on molecular data and fossil calibrations. We compare our time-calibrated phylogeny to temporal patterns of origination and extinction revealed by the Neogene fossil record, and find remarkable congruence between data sets. The timing of events revealed by this analysis strongly implicates paleoenvironmental changes as drivers of diversification in scleractinian corals.

## Results

### Phylogenetic analysis of Caribbean “Faviidae”

We sequenced three single copy nuclear loci for six ingroup and one outgroup Caribbean faviid species. A total of 48 unique alleles were identified for *CaM* (alignment length = 507 bp), 38 alleles were identified for *MaSC-1* (alignment length = 490 bp), and 55 alleles were identified for *Pax-C* (alignment length = 418 bp) (Additional file 1). Maximum likelihood and Bayesian analysis of gene trees showed little support for structure above the species level with no conflict between trees at highly supported nodes (Additional file 2**)**. The taxa *Manicina areolata* and *M. mayori* shared some alleles at all loci, and unique alleles isolated from *Diploria clivosa* and *D. strigosa* did not always form monophyletic groups. A total of 94 individuals with unique genotypes were successfully sequenced at all three loci and used for a concatenated phylogenetic analysis. See Additional file 3 for genotype data of all individuals in study.

**Figure 1:**
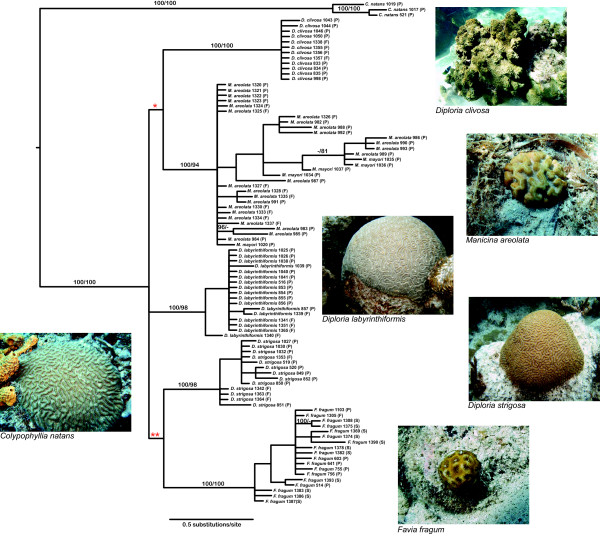
**Phylogenetic tree and photographs of species of the Caribbean Faviidae.** Tree based on a partitioned analysis of individual genotypes at the *CaM, MaSC-1*, and *Pax-C* loci. Terminal taxa are individuals of each species. Letters after sample names indicate coarse geographic sampling information (F = Florida, P = Panama, S = St. Croix). Further sampling and genotype information can be found in Additional file 3. Trees shown were created using Bayesian methods in MrBayes v3.1. Maximum likelihood trees created in RaxML yielded a similar topology. Posterior probabilities (>95%) and bootstrap support (>75%) (Bayesian/ML) are indicated for each node. Dashes indicate nodes unsupported in an analysis. Several deeper nodes in the tree indicated by asterisks were poorly supported in this analysis (* = 72/68, ** = 76/60). Photographs of each species show morphological diversity within this clade. All *Diploria* and *Colpophyllia* species are reef-building, while *Favia* and *Manicina* species are also free-living. (Photo credit: Dr. Charles and Anne Sheppard, http://coralpedia.bio.warwick.ac.uk/)

Bayesian and maximum likelihood trees had identical topologies at all major nodes with support values (Bayesian/ML bootstrap) indicated in Figure 1. The ingroup node was well supported (100/100) as well as species nodes for *C. natans* (100/100), *D. clivosa* (100/100), *D. labyrinthiformi*s (100/98), *D. strigosa* (100/98) and *F. fragum* (100/100). *Manicina mayori* and *Manicina areolata* failed to form monophyletic groups, though support was high at the genus node for *Manicina* (100/94). The genus *Diploria* failed to form a monophyletic group. *Diploria clivosa* formed a clade with *Manicina spp.* and *D. strigosa* formed a clade with *Favia fragum*. Support for these nodes, however, was low (72/68 and 76/60 respectively).

### Timing of divergence

BEAST analysis of the data produced a tree topologically consistent with those of the MrBayes and RaxML analyses. Visual inspection of plots in Tracer v1.5 [41] showed rapid convergence of the analysis and narrowing of priors with all parameters having an effective sample size (ESS) of >1900. The mean rate of substitution was 6.77× 10^-4^ per site (95% Highest Posterior Density (HPD) interval: 4.49 × 10^-4^ - 9.06× 10^-4^) with a coefficient of variation of 1.17 (95% HPD interval: 0.66- 1.72) indicating significant heterogeneity in substitution rates across the tree.

**Figure 2:**
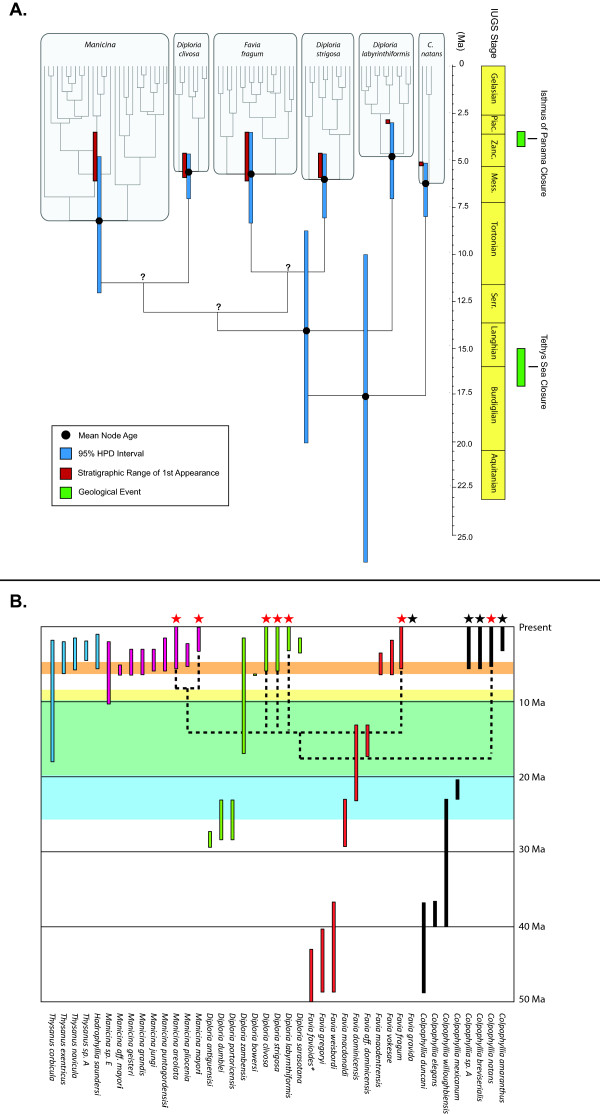
**Caribbean Faviidae chronogram and stratigraphic data.A**) Divergence dates of terminal (species) and internal nodes of a phylogeny of the Caribbean Faviidae. Original chronogram and tree generated in BEAST. Grey boxes indicate species or genera as labeled. Black circles and blue bars correspond to mean node age (Ma) and 95% HPD intervals produced by BEAST analysis. Red bars indicate the stratigraphic age range of the first appearance of that taxon in the fossil record. Green bars next to the time axis are used to indicate major geological events in the isolation of the Caribbean Sea including the closure of the Central American Isthmus at 4.25-3.5 Ma and the closure of the Tethys Sea at 17–15 Ma. Nodes marked with a '?' are poorly supported in this analysis. Detailed information about dates and node calibration can be found in Tables 1 and 2. **B**) Phylogeny on stratigraphy of living and extinct species. Stratigraphic range bars are color-coded by genera, listed on the x axis. Green + blue shading are 95% highest posterior density (HPD) intervals for the ingroup node, and green + yellow shading are 95% HPD intervals for the root node as seen on the chronogram. Orange shading indicates the range of origination dates in the fossil record for all living taxa. Species within genera are ranked by earliest origination date, left to right. The genera *Thysanus* and *Hadrophyllia* are free living, as are all the extinct species of *Manicina*. See Additional file 5 for stratigraphic references. (*Favia favioides range extends to 65.5 Ma – not shown)

Mean ages of species, ingroup, and root nodes with 95% HPD intervals are shown in Figure 2A and listed in Table 1. The posterior mean of the time of the most recent common ancestor (TMRCA) of the *Manicina* group, which was calibrated from species fossil data, shifted several MY from the prior distribution, indicating that the sequence data is influencing divergence dates. For *D. clivosaD. strigosa,* and *F. fragum*, mean estimated ages fell close to the earliest possible dates of their appearance in the fossil record. For the *D. labyrinthiformis* and *Manicina* nodes, fossil dates were closer to the youngest part of the 95% HPD interval. Mean origination time of *D. labyrinthiformis* is pushed back approximately 1.6 MY earlier than previously seen in the fossil record, putting it closer to the origination times of the other species. All mean species origination dates occur shortly prior to the final closure of the Central American Isthmus at 4.25 - 3.45 Ma [11], but we note that the youngest part of 95% HPD for *F. fragum* and *D. labyrinthiformis* overlap with this estimated age of final closure. The timing of the *Manicina* node is considerably earlier than the appearance of the *Manicina areolata* in the fossil record, indicating that this genus diverged earlier than the first appearance of *M. areolata*. Deeper nodes in the tree had significantly larger HPD confidence intervals, due to the lack of fossil calibrations for earlier taxa. The estimate of origination time for the ingroup was 14.10 Ma (95% HPD interval: 8.77-20.09), while origination of the entire Caribbean Faviidae group is indicated by the root node at 17.56 Ma (95% HPD interval: 10.04-26.44). These dates coincide with the timing of the closure of the Tethys Sea in the eastern Mediterranean [10].

**Table 1:** Divergence dates estimated from BEAST

**Taxa**	**Date of Origination - Ma**		
	**Mean**	**Median**	**95% Highest Posterior Density (HPD) interval**
*C. natans*	6.25	6.01	5.16-8.02
*F. fragum*	5.74	5.53	3.52-8.36
*D. clivosa*	5.60	5.41	4.67-7.06
*D. labyrinthiformis*	4.66	4.37	3.01 - 7.06
*D. strigosa*	6.03	5.76	4.67-8.08
*Manicina*	8.21	7.97	4.81-12.08
Ingroup	14.10	13.70	8.77-20.09
Root	17.56	16.86	10.04-26.44

Overlay of the molecular phylogeny onto the fossil stratigraphy reveals three striking patterns (Figure 2B). First, older and extinct *Diploria* and *Favia* cannot be reconciled with this molecular tree, suggesting these genera are not monophyletic. Second, the origination and diversification of a clade of sediment dwelling corals (particularly *Thysanus* and *Manicina*) is confirmed by both the fossil record and molecular phylogeny. Lastly, the appearance of new reef dwelling species of *Favia* and *Diploria* is simultaneous in the fossil record around 5 Ma.

## Discussion

### Phylogenetic relationships within modern Caribbean corals

Thorough sampling of individuals within species in our combined phylogenetic analysis confirms that most modern Caribbean species form well-supported monophyletic lineages (Figure 1). This allows us to reject the idea that widespread hybridization on ecological time scales [42] is important to the evolution of Caribbean faviids, though limited introgression not detected by this data set might have played a creative role in adaptive processes [43]. The exception lies within the two modern species of *ManicinaM. areolata* and *M. mayori*, where extensive allele sharing between species might indicate sub-species status. While *M. areolata* is a seagrass specialist, and drifts remarkable distances on the sediment surface as a free-living adult [44], *M. mayori* is a rare reef species that remains permanently attached as an adult. In Panama, these two morphologically distinct species co-occur within sites yet segregate ecologically by depth-related habitats. An approach that combines ecological/reproductive comparisons, morphometric data, and further genetic analyses such as the coalescent-based model of isolation and migration [45] could resolve this issue.

Above the species level, we could not further resolve the branching order of species within the larger clade. Previous single locus phylogenies using mitochondrial and nuclear genes that have included this group have shown a similar lack of resolution within the Caribbean faviids[35,36]. Another study by Nunes *et al.*[39] shows some supported structure within this group. However, as this paper was looking mainly at broader scale phylogeographic relationships, sampling was done on only few individuals per species within the Caribbean faviids and using only a single mitochondrial marker and a single nuclear marker. For examining relationships below the familial level, the low rates of mtDNA evolution in corals might limit the ability to detect more complex topologies amongst these species. With the increased sampling sizes of multiple loci with higher levels of variation (Additional file 2), we found little evidence for monophyly within genera, and branch lengths tended to be long (Figure 1). Therefore, our inability to resolve relationships among species is consistent with rapid diversification and short internal branch lengths deeper in the tree. With the rapidly declining cost of high throughput sequencing, a phylogenomic approach [46,47] for this set of taxa is likely to improve topological resolution.

### Fossils and molecules reveal the tempo and mode of Caribbean coral diversification

Molecular divergence dating indicates extant Caribbean “faviid” corals radiated rapidly during the late Miocene to early Pliocene (Figure 2). This ecological radiation coincides with a series of biological and physical changes in the structure of shallow marine habitats during the early geological development of the Isthmus of Panama. During the Late Miocene, shallow marine habitats were dominated by broader and more gently sloping sedimentary shelves [48], while productivity in the water column above was much higher compared to the modern productivity of the Caribbean Sea [49]. Klaus *et al.*[15] hypothesize that these extensive mesophotic sedimentary bottoms may have selected for free-living coral species with large tentacle morphologies that were efficient at heterotrophic feeding. Interestingly, our node age for the clade containing the two living *Manicina* species is 8.21 Ma, which coincides with the appearance of other sibling *Manicina* species in the fossil record that have since gone extinct [22]. Thus, it appears we are sampling the evolutionary remnants of a once more diverse and ecologically dominant clade. As the Miocene transitions into the Pliocene, the increasingly isolated Caribbean Sea becomes more oligotrophic and the once broad shelf habitats are now dominated by steeper reef platforms, ideal conditions for primarily photoautotrophic reef species. Our time-calibrated phylogeny shows repeated speciation events of *Diploria* and *Favia* species between ~ 4 – 6 Ma that are either reef specialists or are limited to very shallow (< 5 m) seagrass habitats. Thus the fossil record and molecular data broadly agree on the timing of these ecological radiations, which are temporally correlated with changes in habitat structure and productivity.

Deeper in the tree, node ages for the stem groups of the Caribbean faviids correspond to the isolation from the Mediterranean during the closure of the Tethys Sea (Figure 2A). While these dates support the widely accepted notion of divergence driven by increased isolation of the region, the radiation of the stem group is much later than indicated by the fossil record (Figure 2B). The origination of the *FaviaDiploriaManicina* (FDM) clade is in the early Miocene, but older Oligocene *Diploria* fossils and Eocene *Favia* fossils are more distantly related, suggesting that both genera are para- or polyphyletic. Ken Johnson reached a similar conclusion based on morphological differences [22], hypothesizing that early *Diploria* and *Favia* are unrelated to their modern morphological counterparts. Morphological convergence appears to be a common theme in coral evolution [35] and our analysis points out some of the difficulties in determining the systematic positions of extinct taxa. The use of more informative micro-structural characters that can be quantified in both living and fossil species may be a promising approach to this problem [30].

Congruence of morphology, stratigraphy, and estimates of node ages can be used to include fossil taxa into potentially monophyletic lineages. For example, the diverse members of living and fossil taxa of the genus *Manicina* form a well-supported monophyletic group in Johnson’s morphological phylogeny with all fossil origination dates falling within the lower confidence interval for the molecular *Manicina* node age (Figure 2A). Superimposing the age-calibrated molecular phylogeny onto stratigraphy significantly alters the interpretation of the speed of evolution in this group (Figure 2B), indicating rapid diversification of sediment dwelling corals in the late Miocene.

### Are punctuated patterns driven by adaptation?

Our time calibrated phylogeny confirms fossil evidence that extant Caribbean coral species originated during a period of lineage diversification between 4 and 6 Ma (Figure 2). This diversification event corresponds with ecological radiation into three main ecological niches exemplified by modern Caribbean faviids [21]: (i) small, free living morphologies adapted to sedimentary environments (ii) attached species that live in shallow rubble beds and patch reefs, and (iii) massive colonies the build forereef slopes (Figure 1). During the same period, we also see diversification of reproductive strategies [23], from tightly synchronized annual mass-spawning events and broadcasting larvae typical of *Diploria*[24] to multiple lunar cycles of reproduction and brooding development found in *Favia* and *Manicina*[50,51].

The changes in morphology and life history coupled with widespread environmental changes are suggestive that diversification of Atlantic “faviid” coral might be driven by the evolution of adaptive traits. Using our current phylogeny as a stepping stone, increased genomic and taxonomic sampling of Atlantic corals should allow us to take advantage of several promising new approaches to estimate rates of diversification and evaluate models of adaptive radiation [52,53].

## Conclusions

By combining data from the fossil record with molecular phylogenetic techniques for the first time, this study has given us extensive insight into the tempo of diversification in an ecologically diverse group of Caribbean corals. Two separate lines of evidence now verify the existence of a Mio-Pliocene radiation, while we have been able to additionally confirm species identity, verify origination dates, and understand taxonomic relationships in this diverse and ecologically important group. These findings give us the tools to re-interpret trends seen in the fossil record, allowing us to begin to link patterns of macroevolution to paleoenvironmental changes and gain a new comprehension into the origins and drivers of diversity in the Caribbean.

Besides clarifying evolutionary history, this study has broader contemporary implications. With global change currently causing a rapid decline in coral reef populations around the world [54,55], understanding the processes that generated diversity in coral species will be key to predicting future changes and directing conservation efforts [56]. It has been suggested that species that evolved in a more heterogeneous environment and survived past climatic fluctuations will be more resistant to current global change [29]. Understanding patterns of Caribbean coral evolution during the Pleistocene may be key to understanding the potential outcomes of current environmental impacts.

## Methods

### Taxon sampling

We sampled six of the seven nominal species from the genera *FaviaDiploria*, and *Manicina* that form a monophyletic group within the Caribbean Faviidae [35,39]. The single missing taxon is *Favia gravida*, closely related to *Favia fragum,* but with a distinct non-Caribbean distribution in that it has been only described from Brazil and West Africa [57,58]. We used the genus *Colpophyllia* as the outgroup because it has previously been shown to be a stem taxon to the ingroup species [35,39]. Extensive sampling within each species was conducted at two reef systems in the Caribbean Sea: the Bocas del Toro, Panama, and the Florida Keys, USA with additional *F. fragum* sampled from St. Croix, USVI. The complete list of samples and collection localities is provided in Additional file 3. Skeletal vouchers are deposited in the University of Iowa Paleontology Repository (http://geoscience.clas.uiowa.edu/paleo/index). Samples were collected, preserved, and genomic DNA extracted as described in Carlon and Lippé [59]. Skeletal vouchers were processed by bleaching in a 50% hypochlorite/water solution overnight, rinsing in DI water, and thoroughly drying. Species identification was conducted by D. Carlon in the field and confirmed by A. Budd from vouchers. Complete descriptions of these taxa, photos, and references are available from the Neogene Marine Biota of Tropical American (NMITA) database (http://eusmilia.geology.uiowa.edu/).

### Laboratory protocols

For this study we chose to focus on nuclear markers, since rates of mitochondrial DNA evolution have been shown to be very slow in corals, limiting the ability to detect more recent speciation events [60]. We amplified three single-copy nuclear regions with primers listed in Additional file 4. *Pax-C* and *CaM* primers target introns located within the Pax protein and calmodulin binding protein respectively[61,62], while *MaSC-1* is an anonymous region originally sequenced in *Montastraea annularis*[63]. For PCR amplification of all three loci, we combined: 1 μl of 1x to 100x diluted genomic DNA with a 24 μl PCR master mix consisting of: 0.3 μl of each primer (10 μM), 1 μl dNTPs (2.5 mM each), 2.5 μl 10x reaction buffer, 1 μl MgCl_2_ (25 mM), 1 μl BSA (10 mg/ml), 0.3 μl Taq polymerase (Bioline), and 17.6 μl of H_2_0. Each reaction was run at 95 °C for 10 min, 30 cycles of 94 °C for 30s, Ta for 40s and 72 °C for 1 min, with a final extension of 72 °C for 10 minutes. Purified PCR products were sequenced on ABI 3731 XL 96 capillary DNA analyzers at the University of Hawaii at Manoa and chromatograms were then analyzed and edited using Sequencher 4.5 (Gene Codes). Direct reads revealed indels segregating within many of the species, and preliminary cloning verified multiple indels within all three genes. Since phasing length-variant heterozygotes (LVHs) from direct reads proved unreliable, we cloned 86% of the PCR products from individuals with LVH phenotypes. We cloned PCR products using a TOPO TA cloning kit (Invitrogen) and sequenced using standard M13 primers. Single nucleotide polymorphism heterozygotes were phased using the software PHASE v2.1.1 [64,65]. We used the non-recombination model, and phase thresholds of 0.90. To convert data between FASTA and PHASE formats, we used the webtool SeqPHASE [66]. Haplotype sequence data are available as Genbank PopSets. Accession numbers are listed by species in Additional file 1.

### Phylogenetic analyses

#### Gene trees

Allele sequences were aligned automatically using MAFFT v6 [67,68], and corrected by eye in MacCladev4.08 [69]. Indels were coded as missing data. Models for molecular evolution for Bayesian analysis for both gene trees and the partitioned tree were selected using the Akaike Information Criteria (AIC) in jModelTest v0.1.1 [70,71]. For the gene trees, the best fitting model for the *CaM* and *Pax-C* alignment was GTR + G, and for *MaSC-1* the model was GTR. Bayesian trees for all three loci were generated in MrBayes v3.1 (5,000,000 generations, nruns = 2, nchains = 4) [72,73]. Trees were sampled every 100 generations and 5,000 trees were discarded as burn-in. Maximum likelihood analysis was performed using RaxML 7.2.6 [74,75] with 1000 rapid bootstraps using the default GTR + G model for all loci at the recommendation of the programmers.

#### Partitioned trees

Individuals sequenced at all three loci were used for the construction of a combined partitioned tree. For heterozygotes, SNPs were coded as ambiguous data using standard IUPAC nucleotide ambiguity codes. For the Bayesian analysis, the K80 + G model was chosen for the *CaM* partition, the HKY + I + G model for the*MaSC-1* partitions, and the HKY model for the *Pax-C* partition. Bayesian trees were generated using MrBayes v3.1 (20,000,000 generations, nruns = 4, nchains = 4). Trees were sampled every 1000 generations and 5000 trees were discarded as burn-in. Maximum likelihood analysis was performed using RaxML v7.2.6 with 1000 rapid bootstraps on the Cipres Web Server [76]. For this analysis, the default GTR + G model was used as above.

#### Divergence dating

The program BEAST v1.7.1 [77] was used to estimate divergence dates at species nodes using available fossil data for calibration. Input files for the analysis were generated with Beauti v1.5.4 using a partitioned alignment file of 80 individuals. We used a Yule process speciation prior for branching rates along with an uncorrelated lognormal model for a relaxed molecular clock. Models for molecular evolution for BEAST analysis were selected using the Akaike Information Criteria (AIC) in MrModelTest v2.2 [78]. The HKY + G model was used for the *CaM* and *MaSC-1* partitions, and the HKY + I model for the *Pax-C* partition. Base frequencies were estimated throughout the analysis. Based on the phylogenetic analysis, all species nodes were constrained to be monophyletic except for *M. areolata* and *M. mayori,* which were constrained at the genus node. Shape parameter priors were taken from MrModeltest v2.2 and priors for rates of evolution and Yule birth rates were chosen based on defaults narrowed from preliminary runs.

Stratigraphic ranges of extinct and living Caribbean Faviidae were compiled from the literature and unpublished sources (Additional file 5). Fossil stratigraphic ranges for extant species were used to calibrate species nodes for *Diploria spp., Favia fragum* and *Colpophyllia natans* and the genus node for *Manicina* (Table 2). Calibrations of nodes were done following the guidelines of Ho and Phillips [79]. For all date priors, we used a lognormal distribution with a hard minimum bound set at youngest possible date of first appearance in the fossil record. The mode of the distribution was set to be slightly older than the oldest possible date of first appearance. Finally, the 95% probability distribution was set to encompass a soft maximum bound at the time of the closure of the Tethys (~17 Ma). These distributions incorporate the best-known estimates for origination dates of these taxa, but are wide enough to allow for shifts in dates that may reflect errors due to interpretation or incompleteness of the fossil record.

**Table 2:** Stratigraphic ranges, BEAST calibrations, and section references

**Species**	**Fossil Date of 1st occurrence (Ma)**	**Node Calibration (mean, standard deviation, offset)**	**Median (*95% interval*)**
*C. natans*	5.1-5.3	0.5,1,5.1	6.7 (*5.3-16.8*)
*F. fragum*	3.0-5.6	1.1,0.8,3.0	6.0 (*3.8 - 17.4*)
*D. clivosa*	4.6-5.9	0.6,1,4.6	6.4 (*4.9-17.5*)
*D. labyrinthiformis*	2.9-3.1	0.7,1,2.9	4.9 (*3.2-17.2)*
*D. strigosa*	4.6-5.9	0.6,1,4.6	6.4 (*4.9-17.5*)
*M. areolata*	3.0-5.6	1.1,0.8,3.0*	6.0 (*3.8 - 17.4)*
*M. mayori*	2.9-3.1	n/a*	

Ranges in fossil dates of first occurrence reflect accuracy of section dating. References for dates can be found in Additional file 5. *Calibration for *Manicina is* at genus node since species not well supported in phylogeny.

BEAST was run 4 times (generations = 20,000,000, samplefreq = 1000) on the Bioportal webserver at the University of Oslo [80]. Log files were examined in Tracer v.1.5 [41] to assess convergence of each run. After a 10% burn-in was removed, logs and trees for all runs were combined in LogCombiner v1.7.1 and chronograms were generated with Tree Annotater v1.7.1.

## Competing interests

The authors declare they have no competing interests.

## Authors’ contributions

SAS designed the study, generated molecular sequence data, ran phylogenetic analyses, and drafted the manuscript. AFB confirmed morphological identification of species, compiled stratigraphic data, and helped draft the manuscript. DBC participated in the study design, collected field samples, ran phylogenetic and phasing analyses, and helped draft the manuscript. All authors read and approved the final manuscript.

## Supplementary Material

Additional file 1 **Alleles and Accession Numbers by Species.** Number of individuals sequenced per species (n), the number of alleles isolated per locus per species, and Genbank accession numbers. All species carried unique alleles, except for the two *Manicina* species. The two last rows give the number of unique alleles in the combined *Manicina* data sets (*Manicina spp.*) and the combined 6 ingroup species. (PDF 42 kb) Click here for file

Additional file 2 **Gene trees for (A)*CaM*, (B)*MaSC-1*, and (C)*Pax-C.*** Alleles are designated by locus_allele number. Node labels indicate Bayesian posterior probabilities/Maximum likelihood bootstrap support, -- < 50% ML support. Two-tone boxes indicate alleles shared between taxa. All trees produced in MrBayes v3.1 (generations = 5,000,000, nruns = 2, nchains = 4.) The models of evolution were GTR + G for *Cam* and *Pax-C* and GTR for *MaSC-1.* See Additional File 3 for individual genotypes. Click here for file

Additional file 3 **Sampling and genotype data for all individual corals.** Samples and multilocus genotypes used in gene and concatenated trees. Heterozygous genotypes that could not be resolved by cloning or PHASE 2.1.1 are indicated as ‘?/?+’; and ‘0’ indicates PCR failure or poor sequence quality. The two last columns designate which samples were used in the concatenated ML/Bayes trees (Figure 1) and the BEAST analysis (Figure 2). Click here for file

Additional file 4 **Primers used for direct sequencing.** Sequences and annealing temperatures for primers used in this study. Click here for file

Additional file 5 **Stratigraphic Ranges of the Fossil Caribbean Faviidae.** Compiled first and last occurrence data, references, and notes for all Caribbean fossil faviid species. Click here for file
