# Supplementary material for: Molecules and fossils reveal punctuated diversification in Caribbean “faviid” corals
Source: BMC Evol Biol. 2012 Jul 25;12:123. doi: 10.1186/1471-2148-12-123 (PMC3424149; doi:10.1186/1471-2148-12-123)
Supplement: Additional file 1 — Alleles and Accession Numbers by Species. Number of individuals sequenced per species (n), the number of alleles isolated per locus per species, and Genbank accession numbers. All species carried unique alleles, except for the two Manicina species. The two last rows give the number of unique alleles in the combined Manicina data sets (Manicina spp.) and the combined 6 ingroup species. (PDF 42 kb) [file 1471-2148-12-123-S1.pdf]

**Species**	**n**	***CaM***	**PopSet**	***MaSC-1***	**PopSet**	***Pax-C***	**PopSet**
C. natans	5	4	#XXX	1	#XXX	3	#XXX
D. clivosa	18	4	#XXX	5	#XXX	11	#XXX
D. labyrinthiformis	19	8	#XXX	9	#XXX	9	#XXX
D. strigosa	19	14	#XXX	10	#XXX	14	#XXX
F. fragum	13<br>5	7	#XXX	3	#XXX	6	#XXX
M. areolata	29	10	#XXX	9	#XXX	15	#XXX
M. mayori	5	7	#XXX	5	#XXX	1	#XXX
Manicina spp.	34	15		11		15	
# Unique ingroup alleles		48		38		55	
