# Supplementary material for: Molecules and fossils reveal punctuated diversification in Caribbean “faviid” corals
Source: BMC Evol Biol. 2012 Jul 25;12:123. doi: 10.1186/1471-2148-12-123 (PMC3424149; doi:10.1186/1471-2148-12-123)
Supplement: Additional file 2 — Gene trees for (A)CaM, (B)MaSC-1, and (C)Pax-C. Alleles are designated by locus_allele number. Node labels indicate Bayesian posterior probabilities/Maximum likelihood bootstrap support, -- < 50% ML support. Two-tone boxes indicate alleles shared between taxa. All trees produced in MrBayes v3.1 (generations = 5,000,000, nruns = 2, nchains = 4.) The models of evolution were GTR + G for Cam and Pax-C and GTR for MaSC-1. See Additional File 3 for individual genotypes. [file 1471-2148-12-123-S2.pdf]

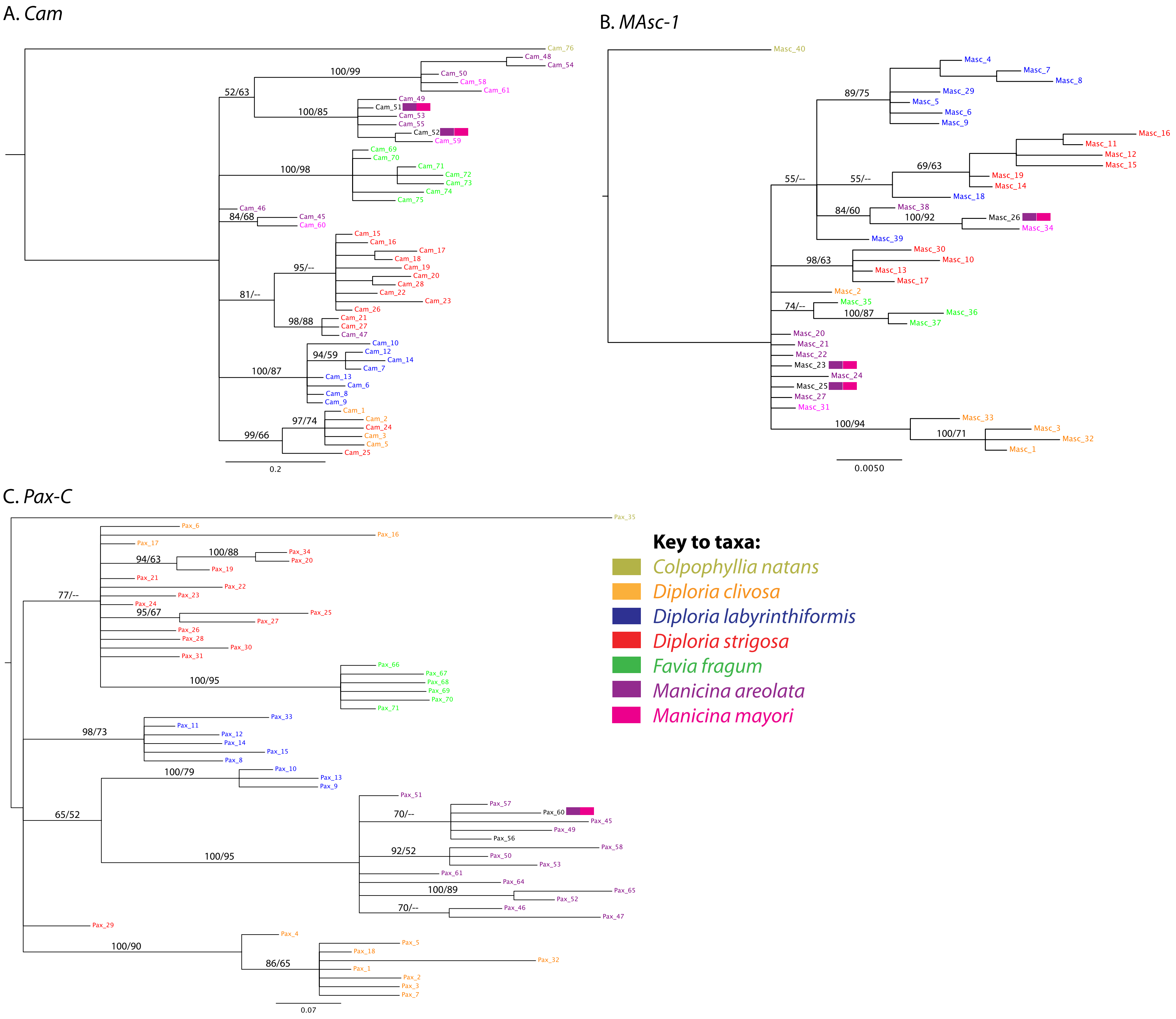
A. Cam
Cam_76
Cam_48
Cam_54
100/99
Cam_50
Cam_58
Cam_61
52/63
Cam_49
Cam_51
100/85
Cam_53
Cam_55
Cam_52
Cam_59
Cam_69
Cam_70
100/98
Cam_71
Cam_72
Cam_73
Cam_74
Cam_75
Cam_46
84/68
Cam_45
Cam_60
Cam_15
Cam_16
Cam_17
Cam_18
95/--
Cam_19
Cam_20
Cam_28
81/--
Cam_22
Cam_23
Cam_26
Cam_21
98/88
Cam_27
Cam_47
Cam_10
94/59
Cam_12
Cam_14
Cam_7
100/87
Cam_13
Cam_6
Cam_8
Cam_9
Cam_1
97/74
Cam_2
Cam_24
Cam_3
99/66
Cam_5
Cam_25
0.2
B. MAsc-1
Masc_40
Masc_4
Masc_7
Masc_8
89/75
Masc_29
Masc_5
Masc_6
Masc_9
Masc_16
Masc_11
Masc_12
69/63
Masc_15
55/--
55/--
Masc_19
Masc_14
Masc_18
Masc_38
84/60
100/92
Masc_26
Masc_34
Masc_39
Masc_30
98/63
Masc_10
Masc_13
Masc_17
Masc_2
74/--
Masc_35
100/87
Masc_36
Masc_37
Masc_20
Masc_21
Masc_22
Masc_23
Masc_24
Masc_25
Masc_27
Masc_31
Masc_33
100/94
Masc_3
100/71
Masc_32
Masc_1
0.0050
C. Pax-C
Pax_35
Pax_6
Pax_16
Pax_17
94/63
100/88
Pax_34
Pax_20
Pax_19
Pax_21
Pax_22
77/--
Pax_23
Pax_24
95/67
Pax_25
Pax_27
Pax_26
Pax_28
Pax_30
Pax_31
Pax_66
Pax_67
Pax_68
100/95
Pax_69
Pax_70
Pax_71
Pax_33
98/73
Pax_11
Pax_12
Pax_14
Pax_15
Pax_8
100/79
Pax_10
Pax_13
Pax_9
Pax_51
Pax_57
70/--
Pax_60
Pax_45
65/52
Pax_49
Pax_56
92/52
Pax_58
Pax_50
100/95
Pax_53
Pax_61
Pax_64
100/89
Pax_65
Pax_52
70/--
Pax_46
Pax_47
Pax_29
Pax_4
100/90
Pax_5
Pax_18
86/65
Pax_32
Pax_1
Pax_2
Pax_3
Pax_7
0.07
Key to taxa:
Colpophyllia natans
Diploria clivosa
Diploria labyrinthiformis
Diploria strigosa
Favia fragum
Manicina areolata
Manicina mayori
