# Supplementary material for: Molecules and fossils reveal punctuated diversification in Caribbean “faviid” corals
Source: BMC Evol Biol. 2012 Jul 25;12:123. doi: 10.1186/1471-2148-12-123 (PMC3424149; doi:10.1186/1471-2148-12-123)
Supplement: Additional file 4 — Primers used for direct sequencing. Sequences and annealing temperatures for primers used in this study. [file 1471-2148-12-123-S4.pdf]

| **Primer** | **Sequence** | **Annealing Temperature ($T_a$)** |
|---|---|---|
| *PaxC-F* | 5' GGA GGA GCT TGC GAA TAA GA 3' | 62 $^{o}$C |
| *PaxC-R* | 5' CCC GGC GAT TTG AGA ACC AAA CCT 3' | |
| *CaM-F* | 5' GGA ACA GTA ATG CGA TCT CTT GGA 3' | 60 $^{o}$C |
| *CaM-R* | 5' TGT CTT TCA TTT TCC GCG CCA TCA 3' | |
| *MaSC1-F* | 5' TGC TGT GAG AAA TAG AAC ACC TG 3' | 60 $^{o}$C |
| *MaSC1-R* | 5' CTG CCA AGA AGG ATC GAT TG 3' | |
